# A Simple Predictive Index of the Abdominal Shape for Postoperative Complications After Laparoscopy-Assisted Distal Gastrectomy for Gastric Cancer

**DOI:** 10.3389/fsurg.2021.768434

**Published:** 2021-12-08

**Authors:** Wei Tao, Yu-Xi Cheng, Xiao-Yu Liu, Bin Zhang, Chao Yuan, Dong Peng, Wei Zhang

**Affiliations:** Department of Gastrointestinal Surgery, The First Affiliated Hospital of Chongqing Medical University, Chongqing, China

**Keywords:** abdominal shape, gastric cancer, laparoscopy-assisted distal gastrectomy, complications, surgical outcomes

## Abstract

**Background:** The purpose of this study was to explore the effect of abdominal shape on the short-term surgical outcomes.

**Methods:** This was a retrospective study that included 425 patients undergoing laparoscopic distal gastrectomy plus D2 lymph node dissection (LADG) from January 2013 to January 2021. The abdominal parameters, including the shortest distance of the pancreas from the anterior abdominal skin (PAAD), the lower sternum angle (LSA), the thickness of the subcutaneous fat at the navel level (SFT), the anteroposterior diameters (APD) and the left-right diameters (LRD) at the navel level, the distance from the xiphoid process to the navel (XND) and the distance from the xiphoid process to the pubis (XBD), were calculated by preoperative abdominal computed tomography (CT) imaging. The parameters and short-term surgical outcomes were analyzed.

**Results:** In males, the number of retrieved lymph nodes was significantly higher in patients with a lower APD group (*p* = 0.031). The operation time was significantly shorter in the lower body mass index (BMI) (*p* = 0.007), lower LSA (*p* = 0.035), lower PAAD (*p* = 0.000), lower SFT (*p* = 0.004), lower APD (*p* = 0.000) and lower LRD (*p* = 0.014) groups. The estimated blood loss was significantly less in the lower BMI (*p* = 0.035), lower LSA (*p* = 0.001), lower PAAD (*p* = 0.012), lower SFT (*p* = 0.003), lower APD (*p* = 0.000) and lower LRD (*p* = 0.005) groups. The complications were fewer in the lower LSA (*p* = 0.012), lower APD (*p* = 0.043) and lower LRD (*p* = 0.023) groups. In females, the postoperative hospital stay was shorter in the lower PAAD (*p* = 0.027) and lower SFT (*p* = 0.004) groups, and the lower SFT group had fewer complications (*p* = 0.020). Furthermore, in multivariate analysis, higher PAAD (*p* = 0.037, odds ratio = 1.030, 95% CI = 1.002–1.059) was an independent factor for predicting postoperative complications in males.

**Conclusion:** Various abdominal shapes can affect the difficulty of LADG. Higher PAAD is a simple independent index for predicting postoperative complications in males.

## Introduction

Gastric cancer is one of the most common malignant tumors in the world, ranking fifth among all tumors (1). The incidence of gastric cancer in Eastern countries is much higher than that in Western countries, especially in China, Japan, and South Korea (2, 3). Laparoscopic distal gastrectomy + D2 lymph node dissection (LADG) has been widely used since its first introduction (4), and the laparoscopic surgical method is widely accepted because of good cosmetic outcomes and it being less invasive than open surgical methods (5).

There are many factors that can affect laparoscopic surgery. Obesity has always been considered a factor that cannot be ignored. Obesity may lead to a decrease in the number of retrieved lymph nodes, prolonged hospital stays, and a poor prognosis (6–8).

In addition to obesity, the abdominal shape is also considered to be one of the factors that may affect laparoscopic surgery. At present, there are various measurements of abdominal shape. Most studies have focused on measurements at the navel level, including the thickness of the subcutaneous fat, the anteroposterior diameters and the left-right diameters, and the area of the visceral fat (9–11). It was found that different abdominal shapes may affect the short-term surgical outcomes of LADG.

Furthermore, the abdominal shape is not only a structure at the navel level but also a three-dimensional structure from the xiphoid process to the pubis. The preoperative evaluation of the abdominal shape might affect the difficulty of LADG; therefore, parameters at other levels are needed. This study focused on some simple abdominal shape parameters and explored their correlations with the short-term surgical outcomes of LADG.

## Materials and Methods

### Patients

A retrospective study of gastric cancer patients who underwent LADG from January 2013 to January 2021 at a single clinical center was conducted in this study. Ethical approval from the Institutional Review Board was obtained (2020-430).

### Surgery

All patients included in this study underwent LADG, and they were operated on by two surgeons with more than 10 years of surgical experience whose learning curve are stable. The surgical methods were all standard LADG, and the scope of lymph node dissection conformed to the Chinese Society of Gastric Cancer guidelines for laparoscopic surgery (12).

### Medical Records Collections

Clinical data were collected from the medical record database. The baseline information included sex, age, BMI, and TNM stage (according to the UICC tumor lymph node metastasis classification system definition). The surgical information included operation time, estimated blood loss, postoperative hospital stay, and the number of retrieved lymph nodes. The postoperative short-term complications included anastomotic leakage, intra-abdominal abscess, pneumonia, anastomotic bleeding, wound bleeding, wound infection, deterioration of liver function, chyle leakage, stenosis, heart disease, gastroparesis, pulmonary embolism, bowel obstruction, cerebral infarction, urinary tract infection, biliary fistula and death within 30 days after LADG according to the Clavien-Dindo classification (13).

### CT Image Data

All patients underwent abdomen computed tomography (CT) examination within 14 days before the operation. The CT images were measured by two physicians who were blinded to the patient's clinical characteristics, and the mean value was calculated. The parameters included the shortest distance of the pancreas from the anterior abdominal skin (PAAD), the lower sternum angle (LSA), the thickness of the subcutaneous fat at the navel level (SFT), the anteroposterior diameters (APD) and the left-right diameters (LRD) at the navel level, the distance from the xiphoid process to the navel (XND), and the distance from the xiphoid process to the pubis (XBD).

**Figure 1:**
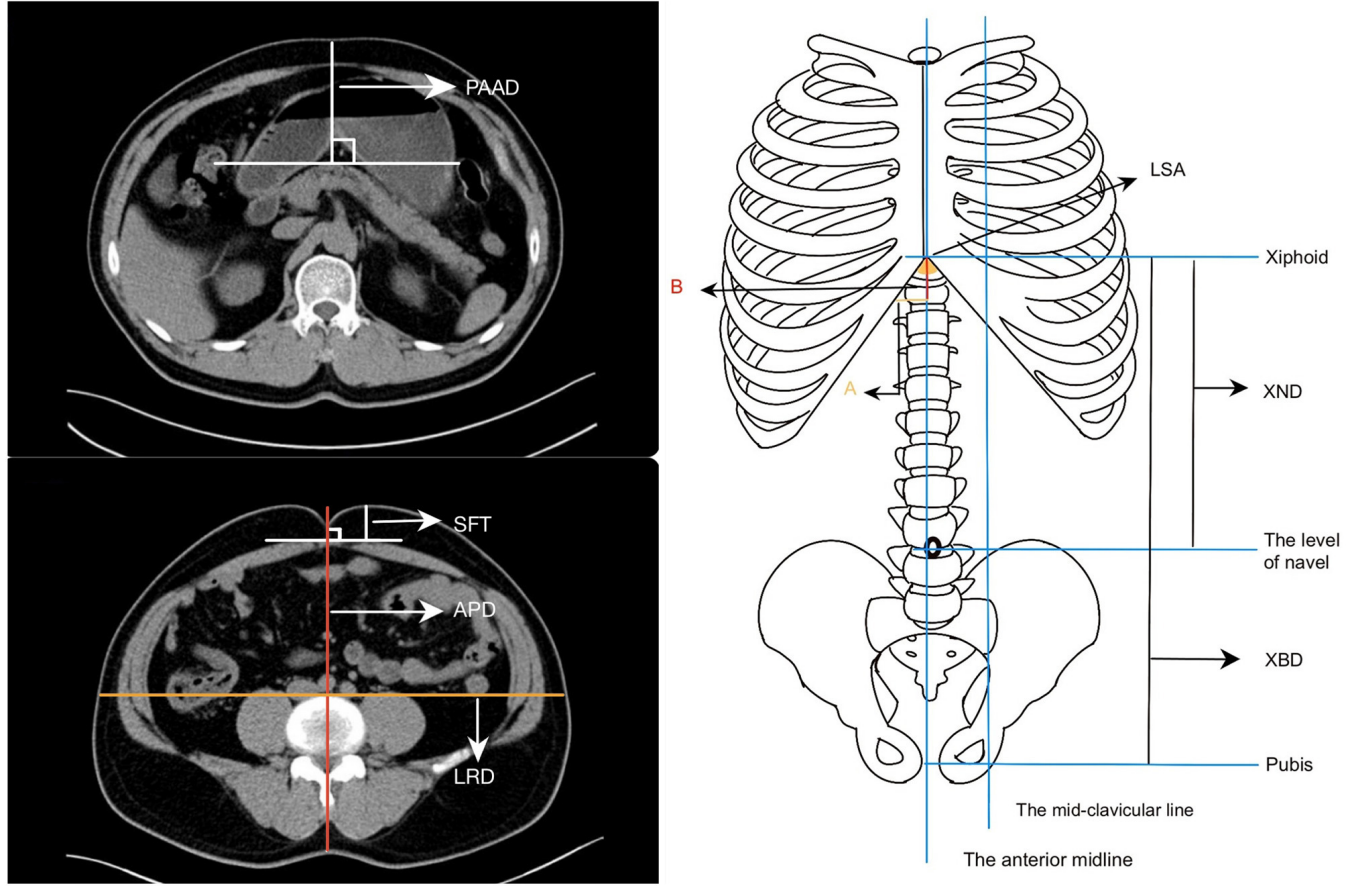
Parameters of the abdominal shape. (a) The shortest distance of the pancreas from the anterior abdominal skin (PAAD). (b) The thickness of the subcutaneous fat at the navel level (SFT), the anteroposterior diameters (APD) and the left-right diameters (LRD) at the navel level. (c) The lower sternum angle (LSA), the distance from the xiphoid process to the navel (XND), and the distance from the xiphoid process to the pubis (XBD).

PAAD was defined as the shortest vertical distance between the pancreas and the skin of the anterior abdomen in the CT image. The LSA was a downward opening angle formed by the combination of the rib arches and the xiphoid process on both sides. It was the apex angle of the triangle formed by the lowermost part of the sternum and the costal arches on both sides, and it could also be called the upper abdominal angle. For calculation of the LSA: take the distance from the anterior midline to the midclavicular line as A and then take the distance from the level at the intersection of the midclavicular line and the costal arch to the xiphoid process as B, after which use the formula LSA = 2^*^DEGREES [ATAN (A/B)] to calculate LSA (14). The relevant data were entered and calculated using Microsoft Excel (2019). At the level of the navel, the distance from the anterior abdomen to the parietal peritoneum was defined as the SFT, the distance from the skin of the navel to the skin of the back was defined as the APD, and the abdominal lateral diameter at the level of the anterior spine was the LRD (Figure 1).

### Statistical Analysis

The median and range were used for continuous variables that did not conform to the normal distribution. The cutoff point of the other continuous variables was taken as the median. The χ2 test, Fisher's exact test or the Mann–Whitney U test were used to test for differences between the two groups. Multivariate analysis was used to detect the independent risk factors for postoperative complications. The data were analyzed using SPSS (version 20.0) statistical software. A bilateral *p*-value of < 0.05 was considered statistically significant.

## Results

### Patients

**Figure 2:**
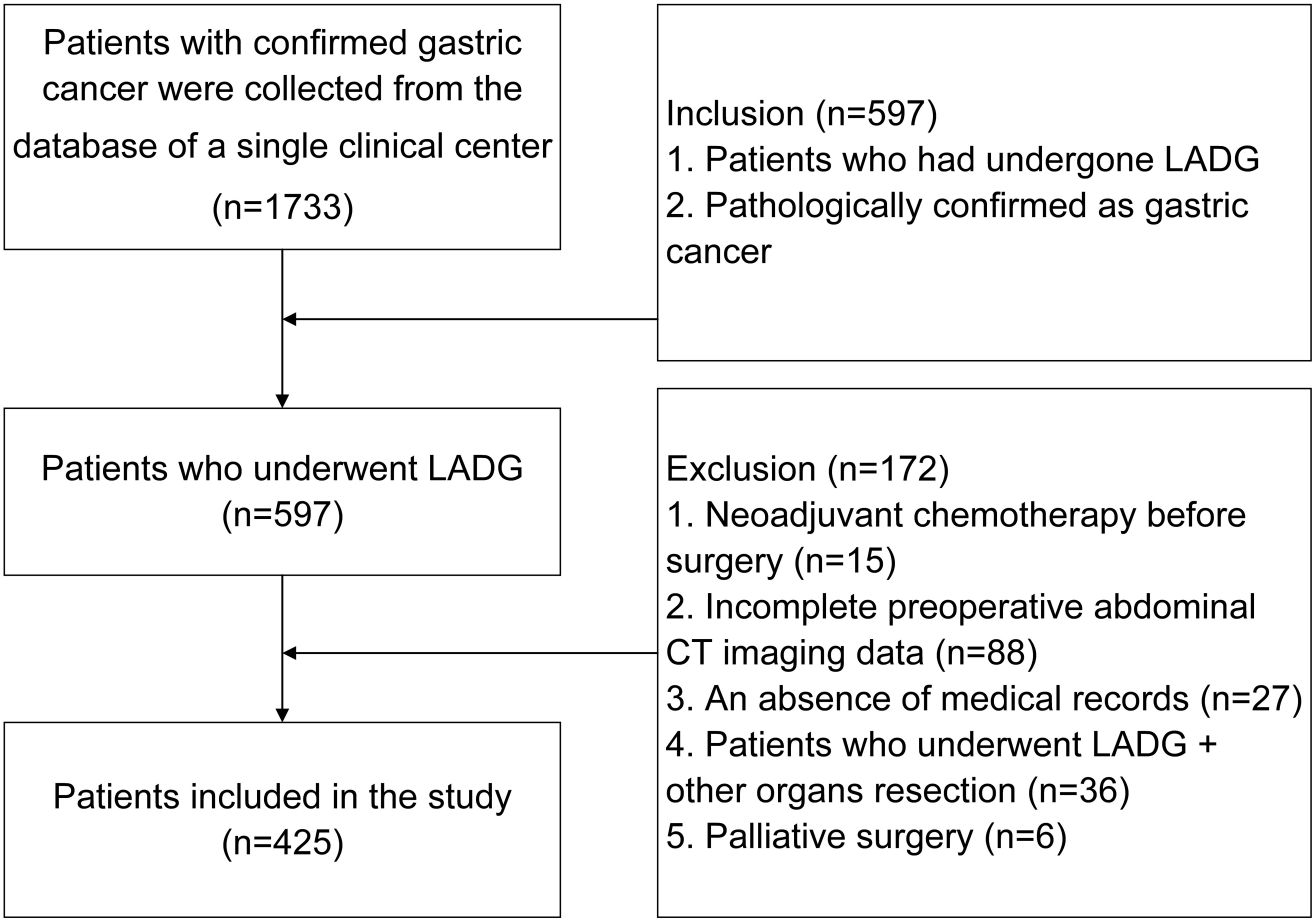
Inclusion and exclusion criteria of patients who underwent laparoscopy-assisted distal gastrectomy.

Patients were included according to the following criteria: 1, patients who had undergone LADG; and 2, pathologically confirmed with gastric cancer. In total, 597 patients were included according to the criteria from among 1,733 patients in the database. The exclusion criteria were: 1, Neoadjuvant chemotherapy before surgery (*n* = 15); 2, Incomplete preoperative abdominal computed tomography (CT) imaging data (*n* = 88); 3, An absence of medical records (*n* = 27); 4, Patients who underwent LADG + other organs resection (*n* = 36); and 5, Palliative surgery (*n* = 6) (Figure 2).

### Clinical Characteristics of the Patients

A total of 425 patients were included in this study, including 318 males and 107 females. The median age was 61 (28–86) years. The median number of retrieved lymph nodes was 22 (5–70). The duration of the operation was 200.0 (85.0–435.0) min. The estimated blood loss was 60.0 (10.0–2,000.0) milliliters. The postoperative hospital stay was 9 (4–66) days. The number of patients with TNM stages I, II, and III were 162, 92, and 171, respectively (Table 1).

**Table 1:** Clinical characteristics of the patients.

**Characteristics**	**No. 425**
**Gender**
Male	318 (74.8%)
Female	107 (25.2%)
Age, years (median, range)	61 (28–86)
**TNM stage**
I	162 (38.1%)
II	92 (21.7%)
III	171 (40.2%)
Retrieved lymph nodes (median, range)	22 (5–70)
Operation time, minutes (median, range)	200.0 (85.0–435.0)
Estimated blood loss, mL (median, range)	60.0 (10.0–2,000.0)
Postoperative hospital stay, day (median, range)	9 (4–66)

*Variables are expressed as the median and range, n (%)*.

*TNM, tumor node metastasis*.

### The Parameters of the Abdominal Shape Between Males and Females

The parameters of the abdominal shape were calculated for males and females. Males had a significantly higher LSA (*p* = 0.001), SFT (*p* = 0.000) and APD (*p* = 0.010) than females. However, there was no difference between males and females in terms of BMI, PAAD, LRD, XND or XBD. Therefore, males and females are discussed separately because of their differences in abdominal shape (Table 2).

**Table 2:** Body shape and BMI of the patients.

**Parameters**	**All (*n* = 425)**	**Male (*n* = 318)**	**Female (*n* = 107)**	***P*-value**
BMI (kg/m^2^)	22.5 (15.0–35.2)	22.5 (15.0–34.5)	22.1 (17.1–35.2)	0.144
LSA (°)	80.9 (43.9–143.0)	82.1 (43.9–143.0)	76.5 (50.1–140.9)	0.001^*^
PAAD (mm)	65.6 (17.6–117.1)	65.2 (20.0–107.4)	66.2 (17.6–117.1)	0.705
SFT (mm)	16.9 (1.0–143.0)	15.6 (1.0–40.5)	21.4 (5.8–143.0)	0.000^*^
APD (mm)	175.1 (122.2–263.0)	178.7 (126.0–263.0)	169.8 (122.2–258.0)	0.010^*^
LRD (mm)	289.3 (206.2–380.8)	289.5 (206.2–380.8)	288.5 (221.0–359.3)	0.669
XND (mm)	215.0 (95.0–340.0)	215.0 (95.0–340.0)	210.0 (165.0–280.0)	0.334
XBD (mm)	370.0 (250.0–525.0)	370.0 (255.0–525.0)	370.0 (250.0–445.0)	0.936

*Variables are expressed as the median and range*,

* *P-value < 0.05*.

*BMI, body mass index; LSA, the angle of the lower sternum angle; PAAD, the shortest distance of the pancreas from the anterior abdominal skin; SFT, the thickness of subcutaneous fat at the navel level; APD, the anteroposterior diameters at the navel level; LRD, the left-right diameters at the navel level; XND, the distance from xiphoid process to navel; XBD, the distance from xiphoid process to pubis*.

### Short-Term Surgical Outcomes in Male Patients

The cutoff point was dichotomized according to the median. The parameters of the abdominal shape were divided into higher and lower groups. The short-term surgical outcomes were compared. In males, the number of retrieved lymph nodes was significantly higher in patients with a lower APD group (*p* = 0.031). The duration of the operation was significantly shorter in the lower BMI (*p* = 0.007), lower LSA (*p* = 0.035), lower PAAD (*p* = 0.000), lower SFT (*p* = 0.004), lower APD (*p* = 0.000) and lower LRD (*p* = 0.014) groups. The estimated blood loss was significantly smaller in the lower BMI (*p* = 0.035), lower LSA (*p* = 0.001), lower PAAD (*p* = 0.012), lower SFT (*p* = 0.003), lower APD (*p* = 0.000) and lower LRD (*p* = 0.005) groups. The complications were lower in the lower LSA (*p* = 0.012), lower APD (*p* = 0.043) and lower LRD (*p* = 0.023) groups (Table 3).

**Table 3:** Short-term surgical outcomes in male patients (*n* = 318).

**Parameters**	**Retrieved lymph nodes**	**Operation time (minutes)**	**Estimated blood loss (mL)**	**Postoperative hospital stay (day)**	**Complications**
**BMI (kg/m** ^**2**^ **)**					
≥22.5 (*n* = 161)	21 (6–48)	210 (85–435)	100 (10–1,800)	10 (4–50)	51
<22.5 (*n* = 157)	22 (6–70)	197.0 (90–395)	50 (20–2,000)	10 (5–62)	35
*P*-value	0.673	0.007^*^	0.035^*^	0.749	0.060
**LSA (** **°** **)**					
≥82.1 (*n* = 159)	22 (6–43)	210 (85–435)	100 (10–1,800)	10 (4–62)	53
<82.1 (*n* = 159)	21 (6–70)	195 (95–380)	50 (10–2,000)	9 (4–33)	33
*P*-value	0.963	0.035^*^	0.001^*^	0.042	0.012^*^
**PAAD (mm)**					
≥65.2 (*n* = 160)	20 (6–48)	215 (85–435)	100 (10–2,000)	10 (4–62)	51
<65.2 (*n* = 158)	22 (6–70)	190 (95–395)	50 (20–500)	9 (4–50)	35
*P*-value	0.335	0.000^*^	0.012^*^	0.284	0.051
**SFT (mm)**					
≥15.6 (*n* = 159)	21 (6–48)	210 (85–435)	100 (10–1,800)	10 (4–62)	47
<15.6 (*n* = 159)	22 (6–70)	190 (95–370)	50 (10–2,000)	10 (4–50)	39
*P*-value	0.165	0.004^*^	0.003^*^	0.932	0.313
**APD (mm)**					
≥178.7 (*n* = 159)	20 (6–48)	213 (85–435)	100 (10–2,000)	10 (4–62)	51
<178.7 (*n* = 159)	23 (6–70)	190 (95–395)	50 (15–500)	9 (6–50)	35
*P*-value	0.031^*^	0.000^*^	0.000^*^	0.221	0.043^*^
**LRD (mm)**					
≥289.5 (*n* = 159)	21 (6–48)	210 (85–435)	100 (10–2,000)	10 (4–62)	52
<289.5 (*n* = 159)	22 (6–70)	190 (90–395)	50 (15–800)	9 (5–50)	34
*P*-value	0.410	0.014^*^	0.005^*^	0.204	0.023^*^
**XND (mm)**					
≥215.0 (*n* = 174)	21 (6–48)	205.5 (90–435)	100 (10–1,800)	10 (4–62)	47
<215.0 (*n* = 144)	22 (6–70)	195 (85–348)	90 (20–2,000)	9 (4–29)	39
*P*-value	0.279	0.141	0.286	0.666	0.989
**XBD (mm)**					
≥370.0 (*n* = 176)	20.5 (6–48)	200 (90–435)	100 (10–1,800)	10 (4–42)	45
<370.0 (*n* = 142)	22 (6–70)	201 (85–375)	100 (20–2,000)	9.5 (4–62)	41
*P*-value	0.223	0.540	0.782	0.933	0.509

*Variables are expressed as the median and range*,

* *P-value <0.05*.

*BMI, body mass index; LSA, the angle of the lower sternum angle; PAAD, the shortest distance of the pancreas from the anterior abdominal skin; SFT, the thickness of subcutaneous fat at the navel level; APD, the anteroposterior diameters at the navel level; LRD, the left-right diameters at the navel level; XND, the distance from xiphoid process to navel; XBD, the distance from xiphoid process to pubis*.

### Short-Term Surgical Outcomes in Female Patients

The parameters of abdominal shape were divided into higher and lower groups in females. The postoperative hospital stay was shorter in the lower PAAD (*p* = 0.027) and lower SFT (*p* = 0.004) groups, and the lower SFT group had fewer complications (*p* = 0.020). However, no difference was found in terms of retrieved lymph nodes, operation time or estimated blood loss (*p* > 0.05) (Table 4).

**Table 4:** Short-term surgical outcomes in female patients (*n* = 107).

**Parameters**	**Retrieved lymph nodes**	**Operation time (minutes)**	**Estimated blood loss (mL)**	**Postoperative hospital stay (day)**	**Complications**
**BMI (kg/m** ^**2**^ **)**					
≥22.5 (*n* = 55)	25 (5–45)	205 (100–330)	50 (10–400)	10 (4–66)	10
<22.5 (*n* = 52)	23 (9–60)	198 (110–310)	50 (10–1,000)	8 (5–51)	9
*P*-value	0.948	0.975	0.307	0.411	0.906
**LSA (** **°** **)**					
≥82.1 (*n* = 54)	23 (5–45)	210 (100–330)	50 (10–1,000)	9 (5–66)	7
<82.1 (*n* = 53)	24 (7–60)	190 (110–303)	50 (10–300)	8 (4–51)	12
*P*-value	0.303	0.220	0.210	0.545	0.190
**PAAD (mm)**					
≥65.2 (*n* = 53)	24 (5–45)	205 (100–330)	50 (10–200)	10 (5–66)	9
<65.2 (*n* = 54)	24 (7–60)	192.5 (110–310)	50 (10–1,000)	8 (4–51)	10
*P*-value	0.295	0.350	0.334	0.027^*^	0.766
**SFT (mm)**					
≥15.6 (*n* = 53)	24 (5–45)	210 (110–330)	50 (10–300)	10 (6–66)	14
<15.6 (*n* = 54)	24 (7–60)	190 (100–310)	50 (10–1,000)	8 (4–35)	5
*P*-value	0.529	0.368	0.777	0.004^*^	0.020^*^
**APD (mm)**					
≥178.7 (*n* = 54)	23 (5–45)	210 (100–330)	50 (10–1,000)	10 (5–66)	11
<178.7 (*n* = 53)	24 (7–60)	195 (110–303)	50 (10–400)	8 (4–51)	8
*P*-value	0.293	0.223	0.143	0.101	0.475
**LRD (mm)**					
≥289.5 (*n* = 54)	23.5 (4–66)	207.5 (100–330)	50 (10–400)	10 (4–66)	13
<289.5 (*n* = 53)	24 (10–60)	190 (110–310)	50 (10–1,000)	8 (5–51)	6
*P*-value	0.221	0.226	0.826	0.119	0.084
**XND (mm)**					
≥215.0 (*n* = 60)	23.5 (5–60)	190 (100–330)	50 (10–1,000)	8 (4–66)	13
<215.0 (*n* = 47)	24 (8–50)	200 (120–315)	50 (10–400)	9 (5–53)	6
*P*-value	0.952	0.297	0.297	0.565	0.232
**XBD (mm)**					
≥370.0 (*n* = 62)	24 (5–60)	190 (100–310)	50 (10–1,000)	8 (5–66)	10
<370.0 (*n* = 45)	22 (7–50)	210 (130–330)	50 (20–300)	10 (4–53)	9
*P*-value	0.385	0.092	0.261	0.131	0.605

*Variables are expressed as the median and range*,

* *P-value < 0.05*.

*BMI, body mass index; LSA, the angle of the lower sternum angle; PAAD, the shortest distance of the pancreas from the anterior abdominal skin; SFT, the thickness of subcutaneous fat at the navel level; APD, the anteroposterior diameters at the navel level; LRD, the left-right diameters at the navel level; XND, the distance from xiphoid process to navel; XBD, the distance from xiphoid process to pubis*.

### Predictive Factors for Postoperative Complications

A total of 105 patients had postoperative complications, and the most common complication was pneumonia (Table 5). Multivariate analysis was used for predicting postoperative complications in males and females, and it was found that higher PAAD (*p* = 0.037, odds ratio=1.030, 95% CI = 1.002–1.059) and older age (*p* = 0.037, odds ratio=1.030, 95% CI = 1.002–1.059) were independent factors (*p* = 0.003, odds ratio=1.047, 95% CI=1.016–1.079) for predicting postoperative complications in males (Table 6).

**Table 5:** Postoperative complications.

**Complications**	**All**	**Male**	**Female**
Anastomotic leakage	20	18	2
Intra-abdominal abscess	9	7	2
Pneumonia	34	31	3
Anastomotic bleeding	9	6	3
Wound bleeding	1	1	0
Wound infection	6	5	1
Deterioration of liver function	1	1	0
Chyle leakage	1	1	0
Stenosis	6	3	3
Heart disease	4	3	1
Gastroparesis	1	1	0
Pulmonary embolism	2	2	0
Bowel obstruction	4	3	1
Cerebral infarction	2	1	1
Urinary tract infection	3	2	1
Biliary fistula	1	1	0
Incisional hernia	1	0	1
Death	0	0	0
Total	105	86	19

*Variables are expressed as n*.

**Table 6:** Multivariate analysis of predictive factors for postoperative complications.

**Risk factors**	**Multivariate analysis**			
	**Male**		**Female**	
	***P* value**	**Odds ratio (95% CI)**	***P* value**	**Odds ratio (95% CI)**
BMI (kg/m^2^)	0.397	1.055 (0.932–1.195)	0.120	0.784 (0.577–1.065)
Age (years)	0.003^*^	1.047 (1.016–1.079)	0.860	1.005 (0.954–1.059)
TNM stage	0.146	1.264 (0.921–1.733)	0.947	1.021 (0.553–1.884)
Pulmonary diseases	0.093	2.277 (0.871–5.951)	0.947	1.118 (0.040–31.066)
LSA (°)	0.256	1.012 (0.992–1.032)	0.168	0.969 (0.926–1.013)
PAAD (mm)	0.037^*^	1.030 (1.002–1.059)	0.217	1.036 (0.980–1.095)
SFT (mm)	0.494	0.981 (0.929–1.036)	0.957	1.002 (0.946–1.060)
APD (mm)	0.894	1.001 (0.983–1.020)	0.347	0.980 (0.939–1.022)
LRD (mm)	0.938	1.001 (0.984–1.018)	0.078	1.030 (0.997–1.064)
XND (mm)	0.328	0.993 (0.979–1.007)	0.831	1.005 (0.962–1.049)
XBD (mm)	0.891	0.999 (0.988–1.011)	0.629	0.993 (0.964–1.023)
Liver diseases	0.999	0.000 (0.000–/)	1.000	0.000 (0.000–/)
Cardiovascular disease	0.598	0.712 (0.201–2.516)	/	/

*Variables are dichotomized according to the median*,

* *P-value < 0.05*.

*BMI, body mass index; LSA, the angle of the lower sternum angle; PAAD, the shortest distance of the pancreas from the anterior abdominal skin; SFT, the thickness of subcutaneous fat at the navel level; APD, the anteroposterior diameters at the navel level; LRD, the left-right diameters at the navel level; XND, the distance from xiphoid process to navel; XBD, the distance from xiphoid process to pubis; OR, odds ratio; CI, confidence interval; TNM, tumor node metastasis*.

## Discussion

This study innovatively explored simple indexes of abdominal shape such as PAAD, LSA and other parameters on short-term surgical outcomes in patients undergoing LADG. We found that abdominal shape can influence the operation time, estimated blood loss, postoperative hospital stay, the number of retrieved lymph nodes and the complications. Furthermore, abdominal body shape differed between sexes, males had larger APDs and smaller SFTs than females. Therefore, we analyzed males and females separately and found that few abdominal shape parameters had an impact on short-term surgical outcomes in females. However, we found that some abdominal shape parameters were crucial in males. This result was consistent with the previously published literature (10, 11, 15), the probable reason accounting for the difference between males and females might be that: men accumulated more visceral fat, whereas women accumulated more subcutaneous fat (10).

Obesity has always been considered an independent factor in patients with gastric cancer. BMI can be a parameter to represent obesity. Some studies believe that BMI has an impact on postoperative complications and survival (16, 17). In this study, males with a lower BMI had a shorter operation time and a smaller estimated blood loss. Obesity has an impact on the difficulty of performing LADG in male patients.

The operation time and estimated blood loss can reflect the difficulty of the operation. Previous studies have found that a large APD can increase the operation time and that a large LRD can increase the amount of surgical bleeding (10, 11). In this study, a larger PAAD, LSA, SFT, APD and LRD contributed to an increase in the operation time in males. Male patients with larger LSA, PAAD, APD, SFT and LRD could experience larger amounts of estimated blood loss, but all parameters had a negative relationship in females. Different distensibilities of the abdominal wall in males and females due to the accumulation of muscle and lipids may explain why the abdominal shape was more strongly associated with male patients than female patients (18). The extra operation time and blood loss may be due to the large size of the abdominal shape and excess fat tissues accumulating around vessels (11). However, a larger LSA resulted in a larger amount of estimated blood loss in males, and the cause remained unclear because the LSA is made up of the xiphoid and the rib arch, and it is relatively fixed. A previous study reported that the LSA could be a simple predictive index for a larger estimated blood loss during laparoscopy-assisted total gastrectomy. The possible reason for this is that a larger LSA could cause the five trocar sites to be closer, and the instruments might then interfere with each other, which increases the difficulty of the operation and might cause more blood loss (14). We considered that the LSA may increase the space in the abdominal cavity, leading to difficulty and extra blood loss.

Previous studies reported that postoperative complications ranged from 6.1 to 30% (5, 11, 19, 20). These studies focused on the associations of BMI and visceral fat with postoperative complications (21, 22). In this study, the rate of postoperative complications was 24.7%, a higher PAAD was an independent factor for predicting postoperative complications in males. This might be because of the large size of the abdomen that affects the safety of LADG, so surgeons should be cautious about operating on patients with a higher PAAD.

The number of retrieved lymph nodes is related to the effectiveness of the operation. An insufficient number of retrieved lymph nodes may lead to a poor prognosis (23, 24). In this study, males with a lower APD had more retrieved lymph nodes, and it seems that smaller parameters of the abdominal shape can make LADG surgery more effective. Therefore, much attention should be given to patients with larger parameters of the abdominal shape.

This study has some limitations as well. First, this is a retrospective study in a single center, therefore, multicenter and high-quality studies are needed in the future. Second, this study only included patients undergoing LADG, and there may be some patients who underwent open gastrostomy because of obesity or were converted to open gastrostomy due to the large amount of bleeding or the difficulty of LADG. Third, this study used CT to estimate the LSA. Ideally, we think of the rib arch as a straight line, but actually, the rib arch is a curve, and some patients have asymmetrical bilateral costal arches. The calculation of the angle in this study may have some deviations.

In conclusion, various abdominal shapes can influence the difficulty of LADG. A larger PAAD is an independent factor for predicting postoperative complications in males.

## Data Availability Statement

The original contributions presented in the study are included in the article/supplementary material. Further inquiries can be directed to the corresponding author.

## Ethics Statement

This study was conducted in accordance with the World Medical Association Declaration of Helsinki and was approved by the Medical Ethics Committee of the First Affiliated Hospital of Chongqing Medical University (2020-430). Written informed consent for participation was not required for this study in accordance with the national legislation and the institutional requirements. Written informed consent was not obtained from the individual(s) for the publication of any potentially identifiable images or data included in this article.

## Author Contributions

WT: conceptualization, formal analysis, software, and writing-original draft. Y-XC: data curation, formal analysis and investigation, and resource. X-YL, BZ, and CY: data curation, project administration, and resources. DP: conceptualization, project administration, supervision, and writing—review and editing. WZ: conceptualization, supervision, and writing—review and editing. All authors contributed to the article and approved the submitted version.

## Conflict of Interest

The authors declare that the research was conducted in the absence of any commercial or financial relationships that could be construed as a potential conflict of interest.

## Publisher's Note

All claims expressed in this article are solely those of the authors and do not necessarily represent those of their affiliated organizations, or those of the publisher, the editors and the reviewers. Any product that may be evaluated in this article, or claim that may be made by its manufacturer, is not guaranteed or endorsed by the publisher.

## Abbreviations

LADG: The surgery of laparoscopic distal gastrectomy + D2 lymph node dissection
PAAD: The abdominal parameters including the shortest distance of the pancreas from the anterior abdominal skin
LSA: the lower sternum angle
SFT: the thickness of subcutaneous fat at the navel level
APD: the anteroposterior diameters at the navel level
LRD: left-right diameters at the navel level
XND: the distance from xiphoid process to the navel
XBD: the distance from xiphoid process to the pubis.
